# Quantitative molecular imaging of angiogenesis-targeted fluorinated nanoparticles: new approaches for B_1_-mapping compensation for ^19^F-MRI

**DOI:** 10.1186/1532-429X-15-S1-O83

**Published:** 2013-01-30

**Authors:** Matthew J Goette, Anne H Schmieder, Todd A Williams, John S Allen, Jochen Keupp, Gregory M Lanza, Samuel A Wickline, Shelton D Caruthers

**Affiliations:** 1 School of Medicine, Washington University in St. Louis, St. Louis, MO, USA; 2 Philips Research Europe, Hamburg, Germany

## Background

Quantitative MR molecular imaging allows for the detection of targeted contrast agents to diagnose disease states and monitor response to therapy, such as anti-angiogenic therapy in atherosclerosis and cancer with α_ν_β_3_-integrin targeted perfluorocarbon (PFC) nanoparticles. Recently, ^19^F MR using a ^19^F/^1^H dual-tuned RF coil has been utilized to directly image and quantify the fluorinated core of these PFC nanoparticle (NP) emulsions. However, low concentrations of these fluorine agents in the body, in conjunction with varying RF coil sensitivity profiles (B_1_-field inhomogeneities) raise obstacles to accurate quantification. This study presents a strategy to more accurately quantify the sparse ^19^F signal from PFC NP emulsions with a ^1^H image-based Actual Flip-angle Imaging (AFI) B_1_-mapping correction to the ^19^F and ^1^H images.

## Methods

New Zealand White Rabbits (2 kg) were implanted with a VX2 adenocarcinoma tumor (2-3 cm) in the hind leg. Angiogenesis imaging was performed 2 weeks post implantation, under ketamine/xylazine anesthesia. An α_ν_β_3_-integrin targeted perfluoro-octyl bromide (PFOB) nanoparticle emulsion was prepared, and injected intravenously 3 hours before imaging. MR data were acquired on a 3T clinical whole-body scanner (Achieva, Philips Healthcare) with a dual ^19^F/^1^H spectrometer system and a dual-tuned transmit/receive single loop surface RF coil (7×12 cm). A simultaneous ^19^F/^1^H gradient echo (GRE) imaging sequence was used with: ^19^F offset frequency on the center of the PFOB CF_2_ peak, 15 4-mm slices, 140 mm FOV, 48^3^ matrix, α = 60°, TE/TR = 2.2/8.5 ms, 21 min scan time. The B_1_ field was mapped using an AFI sequence with matching geometry. Using the flip angle map and a model of the GRE signal, a spatially-dependent calibration mask was calculated in MATLAB (MathWorks) and used to compensate the ^1^H and ^19^F signal intensities for the GRE sequence.

## Results

**Figure 1:**
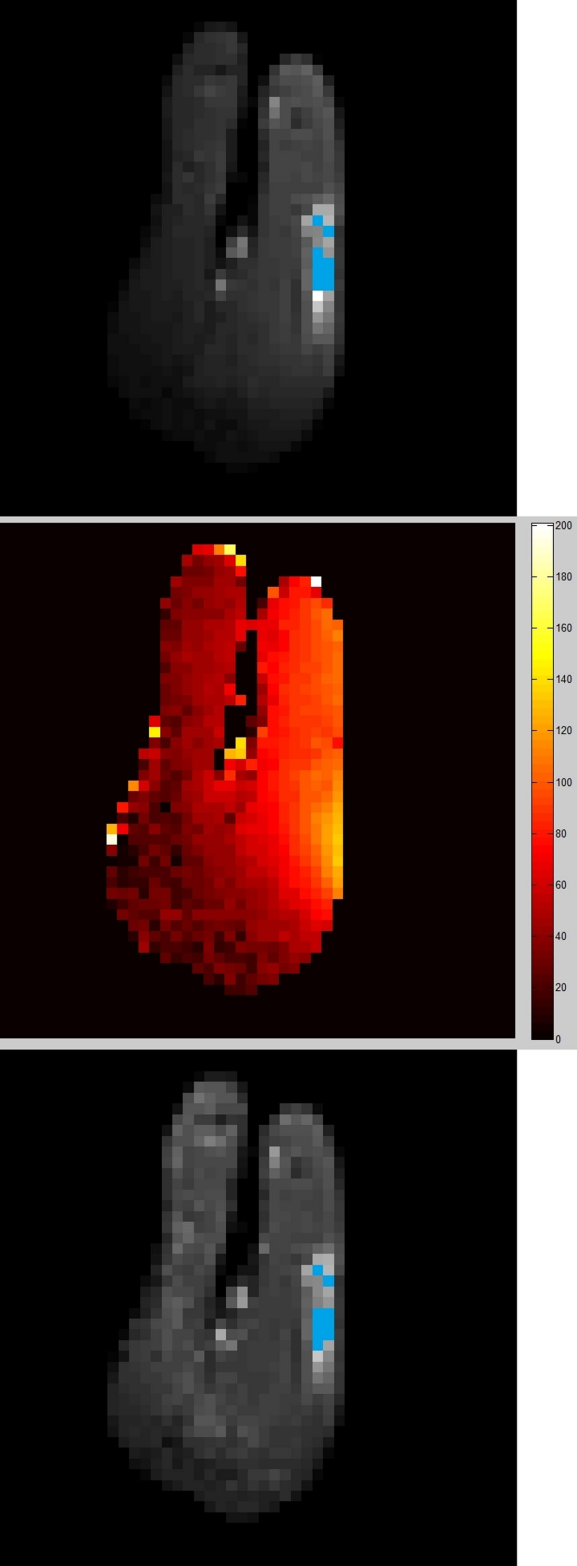
Top: uncorrected ^1^H image of rabbit model with angiogenesis-targeted ^19^F imaging (blue overlay). Middle: AFI B_1_ map (% actual/requested flip angle). Bottom: ^1^H and ^19^F superimposed images using AFI B_1_-mapping correction.

PFC NP targeted the tumor neovasculature, and provided localized ^19^F signal as expected. Figure 1 displays the uncorrected (top) and corrected (bottom) ^1^H images with the ^19^F signal superimposed, using the AFI (middle) B_1_-mapping correction technique. After correction, the ^1^H signal intensity profile as a function of distance from the surface coil (located at right) is improved. After the same correction to the ^19^F signal, the measured concentration of nanoparticles when compared to a standard was 10.2 ± 1.0 mM_19F_, versus 9.0 ± 2.2 mM_19F_ before correction.

## Conclusions

An image-based B_1_-mapping correction acquired with ^1^H can be used to correct signal intensities for ^19^F and ^1^H images of angiogenesis in an *in vivo* rabbit model. The correction results in a more homogeneous ^1^H image of the anatomy and facilitates accurate measurement of bound α_ν_β_3_-integrin targeted nanoparticles with ^19^F imaging.

## Funding

AHA 11PRE7530046; NIH R01 HL073646.

